# True-3D Accentuating of Grids and Streets in Urban Topographic Maps Enhances Human Object Location Memory

**DOI:** 10.1371/journal.pone.0116959

**Published:** 2015-02-13

**Authors:** Dennis Edler, Anne-Kathrin Bestgen, Lars Kuchinke, Frank Dickmann

**Affiliations:** 1 Department of Geography, Ruhr-University Bochum, Bochum, Germany; 2 Department of Psychology, Ruhr-University Bochum, Bochum, Germany; Birkbeck, University of London, UNITED KINGDOM

## Abstract

Cognitive representations of learned map information are subject to systematic distortion errors. Map elements that divide a map surface into regions, such as content-related linear symbols (e.g. streets, rivers, railway systems) or additional artificial layers (coordinate grids), provide an orientation pattern that can help users to reduce distortions in their mental representations. In recent years, the television industry has started to establish True-3D (autostereoscopic) displays as mass media. These modern displays make it possible to watch dynamic and static images including depth illusions without additional devices, such as 3D glasses. In these images, visual details can be distributed over different positions along the depth axis. Some empirical studies of vision research provided first evidence that 3D stereoscopic content attracts higher attention and is processed faster. So far, the impact of True-3D accentuating has not yet been explored concerning spatial memory tasks and cartography. This paper reports the results of two empirical studies that focus on investigations whether True-3D accentuating of artificial, regular overlaying line features (i.e. grids) and content-related, irregular line features (i.e. highways and main streets) in official urban topographic maps (scale 1/10,000) further improves human object location memory performance. The memory performance is measured as both the percentage of correctly recalled object locations (hit rate) and the mean distances of correctly recalled objects (spatial accuracy). It is shown that the True-3D accentuating of grids (depth offset: 5 cm) significantly enhances the spatial accuracy of recalled map object locations, whereas the True-3D emphasis of streets significantly improves the hit rate of recalled map object locations. These results show the potential of True-3D displays for an improvement of the cognitive representation of learned cartographic information.

## Introduction

Object location memory is an essential aspect in orientation and navigation tasks. When learning object locations, people individually reorganise the structure of a spatial configuration to develop a cognitive map of the represented topography [1]. Such cognitive maps are deduced from the environment itself (direct experience / primary learning) or from the use of external sources providing spatial details about the environment (indirect experience / secondary learning). These external references can be verbal descriptions [2–4] or cartographic media, i.e. maps or map-like displays [5–7]. Interdisciplinary studies, at the intersection of cognitive psychology and cartography, provided evidence that spatial information acquired from map sources is substantially modified on its way from the original reference to the recall from memory. Original information is distorted in many cases. These distortions follow reproducible phenomena (for overviews, see [1, 8–11]). For instance, it was shown that systematic distortions rely on a hierarchical coding of spatial information and configurations [12–15]. Hierarchical memory coding refers to nested levels of detail including superordinate as well as subordinate structure units. A subsequent hierarchy in memory, from bigger to smaller units, has an impact on spatial judgements. A single map object is learned as part of a superordinate reference structure [16]. The majority of distortions in spatial cognition are related to perception-based phenomena that reflect principles of perceptual grouping (Gestalt laws) [17,18]. Thus, human spatial memory is affected by manipulations of Gestalt illusions, such as common region, connectedness or symmetry [12, 18–22]. These studies, like most other studies on indirect learning of spatial information, did not use maps as materials, but simple map-like layouts that did not include detailed representations of geographic environments in the topographic base layer. The results of these studies, however, show that graphical alterations affect the quality of the user’s mental representation. This invites mapmakers and cartographers to identify, explore and utilise those design options that can help to improve map perception and spatial memory, and thus support the formation of a more accurate cognitive representation of the environment [8, 23].

The perceptual organisation of a spatial layout is known to have an early beginning [9,24] and likely starts with the first fixation of the viewer [25–27]. It is suggested that both perception- and memory-based processes influence spatial memory in a conjoint manner [28]. The reader breaks up the map face into a set of object divisions (“spatial chunks”). Such perception-based chunking is guided by structuring map elements that regionalise the map surface. These elements can promote the learning of objects and their spatial relations [12, 19, 29]. Here, learning is related to a superordinate reference framework consisting of specific graphic elements, such as artificial coordinate grids and content-related features like road, river and railway systems [16, 30, 31]. These graphic elements establish a functional structure for map perception and memory. They represent contents and, at the same time, they support perception of information, as they create an optimised reference of map objects to the topographic feature they represent. These spatial information aggregates are also structured in a hierarchical fashion and are as such essential components of cognitive representations [14, 16, 21].

Recent studies on vision research and spatial memory show that chunking elements improve human memory performance. It was shown that artificial, structuring elements, such as continuous grid lines, support the perception and recognition of object locations in images [32–35]. Grids, which are traditional cartographic elements used to locate represented map objects or to define their coordinates [36–38], also increase performance in map-based object location memory tasks [30, 39, 40]. Of interest is that the effects of grids interact with the complexity and geographic context represented in the topographic base map. The positive effect of grids for object location memory was strong in different topographies. Its strength however decreased in urban landscapes [30], which calls for new design approaches to increase the effects, particularly in urban topographies.

In recent years, True-3D representations, such as lenticular visualization based on autostereoscopic displays, have broadened the spectrum of analogue and, especially, digital cartography [41]. In the theory of 3D imaging techniques, a distinction is made between Pseudo-3D and True-3D visualisations [42]. The term Pseudo-3D refers to visualizations on a planar media based on a monoscopical perspective. The viewer gets a 3D impression due to the graphic perspective of a single image. The term True-3D refers to depth illusions based on a stereoscopic visualisation of (at least) two images [43–46].

The increase of True-3D displays as products of mass media has been huge. Based on autostereoscopic displays, True-3D vision is now available without any additional devices, such as polarised glasses, shutter glasses or red and green glasses [46]. Autostereoscopic displays allow cartographers to design True-3D maps including several information layers that are anchored at different depth positions. Thus, some map information can appear closer to the viewer than other parts of the map, which may have effects on the user’s attention and information processing [47, 48].

In early research on visual perception in cartography, it was suggested that those information planes overlaying other map information get a higher significance in map perception and, accordingly, create a visual hierarchy from the top to the bottom layer [49]. So far, relatively few studies have examined visual attention and information processing in 3D stereoscopic contexts [50, 51]. Based on eye-movement registration, it was demonstrated that image structures catching the attention of the viewers are the ones projected closer towards them [52]. It was shown that information projected closer to the viewer was being fixated earlier than information in far regions [53, 54]. Moreover, 3D stereoscopic content led to an increased spatial extend of image exploration, to an increased number of fixations and to shorter and faster saccades [53] and shorter fixation durations [55]. These results lead to the assumption that content accentuated in depth might guide the deployment of attention [56]. It seems apparent that the use of true-3D brings advantages for information processing. Exploring the opportunities of autosteroscopic displays in cartography, it was recently shown that the use of different z-positions can improve information processing. Maps are read significantly faster when map symbols are positioned in different depth layers [57].

In terms of spatial memory tasks, no references were found that reported empirical studies based on 3D stereoscopic content. With reference to static images including Pseudo-3D depth effects, it was earlier shown that locations of alphabetic letters are recalled more accurately [58]. The results of this study indicated a correlation between spatial memory and 3D graphical user interfaces [59]. However, it has not yet been explored whether using stereoscopic depth could bring an advantage for human object location memory in maps. Based upon the results of the literature compiled above [50–57], it can be assumed that true-3D depiction of chunking linear elements in maps would attract the map user’s attention. It thus can be expected that a greater visual attention to the chunking structures in maps, such as grids and streets, improves human object location memory.

This paper reports the effects of the True-3D representation of linear map features in urban topographic maps on object location memory, in comparison to ‘traditional’ 2D maps with no change in depth. Two studies are conducted to explore the True-3D effects of grids (Study 1: 3D vs. 2D Grids) and streets (Study 2: 3D vs. 2D Streets). Following the results of previous studies [8, 19, 30, 40], it is assumed that the systematic structure of grids and the pattern of the streets divide the map surface into information aggregates, which enhances the spatial memory performance. The 3D depth effect (grid / street layer appears closer to the viewer) is expected to guide the user’s attention to the graphically emphasized orientation pattern provided by the grids and lines [50–57], and, thus, to support the user’s object location memory. Using a standard approach to investigate spatial memory the participants are asked to recall previously studied object locations learned from urban topographic maps (recall paradigm; see also [14, 22, 60–63]. The recall performance is measured by the percentage of correctly recalled object locations (hit rate) and their mean distances from the original object location (spatial accuracy).

## Study 1: 3D vs. 2D Grids

### Methods

The study was conducted in accordance with the Declaration of Helsinki and approved by the local ethics committee of the Faculty of Psychology, Ruhr-University Bochum (Germany). All participants gave their written informed consent before inclusion in the study.

#### Participants

Sixty-eight participants (33 female, 35 male) aged between 20 and 32 [M = 24.7; SD = 2.6] took part in the study for pay. All participants were unaware of the study purpose and reported having normal vision or corrected-to-normal vision. All participants were students at the Ruhr-University Bochum (RUB). They were unfamiliar with the topography represented in the study materials.

#### Materials

14 different digital topographic maps were created as study materials. The maps were derived from *ATKIS-Basis-DLM*, the topographic geodata base acquired and maintained by German public authorities. They considered all layers of the present digital topographic map of North Rhine-Westphalia, Germany (DTK10-NRW). Any verbal elements, such as written place names and other object / attribute labels, were removed from the maps to avoid connotations. All map sizes were 1208 px * 805 px (30 cm * 20 cm). The scale of each map was 1/10,000. The user’s viewing distance from the screen was 50 cm.

The 14 maps were designed according to two conditions—seven maps in 2D, seven maps in True-3D. One map of each condition was assigned to a practice trial. Each map represented a highly urban topography (Ruhr district) of about the same map complexity. The complexity was measured in total number of distinct objects (DOs) using a multiresolution segmentation algorithm [64] and further image classification methods implemented in the geographic object-based image analysis (GEOBIA) software *Trimble eCognition Developer 8.7.2*.

**Fig 1:**
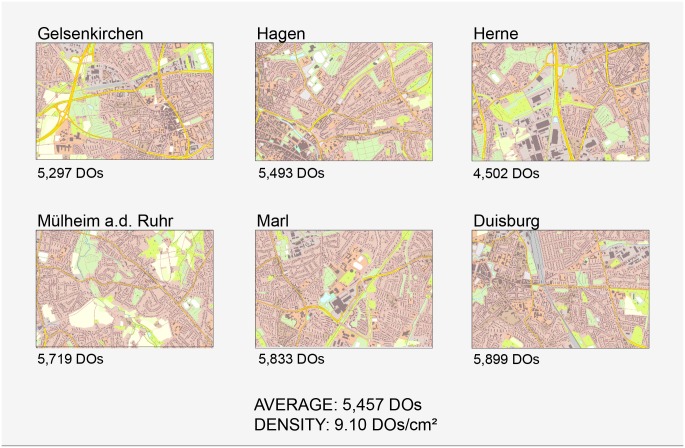
The six highly urban maps used in Study 1. Each map (scale 1/10,000) was derived from *ATKIS-Basis-DLM* and represented the characteristic highly urban topography of the Ruhr district, Germany (see place name labels above the maps). The distinct objects (DOs) of each map were determined as quantifying measure of map complexity. The average number of DOs and the object density (Average DOs / 600 cm²) were additionally determined.

**Fig 2:**
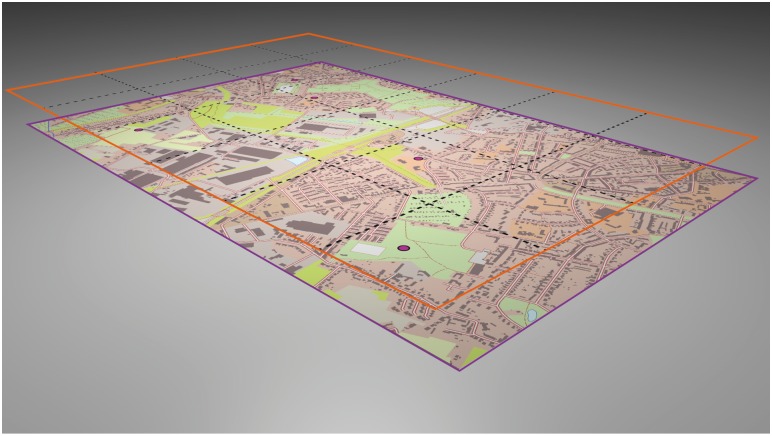
Oblique bird’s eye view on a study-test map, incl. True-3D accentuated grids. The figure indicates the layer arrangement of the two different elevation levels—highly urban topographic base map (violet frame; cf. map of Gelsenkirchen, Fig. 1) and layer of rouletted grids (orange frame). Both layers were projected in a 30 cm*20 cm format. The depth offset between both layers was 5 cm. The five point symbols in the topographic base map represent the randomly selected places of interest (POIs) to be learned and recalled. The thickness of the grid lines was scaled up in this figure to better illustrate the grid construction. The original gauge was 0.5 pt (0.18 mm).

In the 3D maps, the grid layer was accentuated by raising its position into the z-dimension. Using a lenticular lense mask mounted at the display panel, the streets hovered over the base map and appeared closer to the user than in the 2D maps where all layers had the same depth position. Fig. 1 gives an impression of the six maps used in the study-test trials for 2D and 3D. All maps were additionally featured with a “rouletted grid” [38], i.e. a grid construction made up of dashed lines (see also [30]). For technical reasons (i.e. the arrangement of the lense foil mounted at the display), the more common grid consisting of continuous lines was not possible to visualise in 3D. Based on previous findings in object location memory [39], the grid line separation was 5 cm. The selected separation of 5 cm divided the map surface (30 cm * 20 cm) into 24 grid cells, each of which comprised 25 cm². The depth offset between the topographic base layer and the grid layer was 5 cm. Fig. 2 gives an impression of a study-test map including an elevated grid construction.

The maps were additionally featured with five circular symbols representing the locations of fictional places of interest (POIs). The five POIs were randomly selected from a set of 50 objects previously defined for each map. Care was taken not to position POIs onto the grids. All symbols were identical in size (d = 1 cm) and colour (R: 225, G: 0, B: 200). *The study maps were created using ArcMap 10*.*1*, *Adobe Illustrator CS 5* and *Cinema 4D*. The final maps were embedded into a tool based on *Adobe Flash CS 5*. This tool was used to run the trials and to acquire all test data needed. The maps were displayed on a Full HD LED screen (*Samsung SyncMaster SA300*, 22”) that was calibrated in order to project the official map colours.

#### Procedure

The study consisted of a between-subject design including the factor GRIDS (3D/ 2D). The sixty-eight participants were randomly assigned to one of the two groups. As about 2–5% of people show stereo-anomalous or stereo-blind performance [65], it was necessary to apply filter questions. These filter questions referred to a digital collage presented on the 3D display. This collage comprised several images projected in different depth layers. It was used to check whether the participants were able to perceive the 3D effects caused by the lenticular display. Whenever people had problems with properly realising these effects, they were excluded from the study. Two additional participants did not pass this preliminary check. Each of the sixty-eight participants took part in three study-test trials in random order. In these trials, they were shown one of the three study maps for 45 s and were asked to learn the locations of five randomly distributed places of interests (POIs). The study phase was immediately followed by a filler task (45 s of multiplication exercises). Finally, the map was shown again for 45 s without POIs, and the participants were requested to recall the five POI locations. To locate the recalled POIs within the recall task, the participants were instructed to use the mouse cursor. The participants were allowed to shift the location of each POI until they confirmed the final recall position using a keyboard command. Before the start of the three study-test trials, the participants were given a practice trial to become familiar with the software, the tasks, and the general test procedure. The participants were encouraged to complete their tasks as quickly and accurately as possible.

#### Analyses

The recall performance was assessed by measuring the Euclidian distance between the x- and y-coordinates of the recalled POIs and the corresponding coordinates of their original position on the map. The distance was measured in pixels (px). In accordance with previous research [8, 22, 30, 39, 40], the location of a recalled POI was considered correct if it deviated no more than 40.27 px (0–1 cm) from the location of the object to be learned. An independent-measures t-test was applied to compare the means from the 2D- and 3D-GRID conditions. It was computed for the hit rate (percentage of correctly recalled POIs) and spatial accuracy (mean distances of correctly recalled POIs). The significance threshold was set at p = .05. Only responses were considered for the statistical analyses when at least one correctly remembered POIs contributed to a map average per participant. Due to this constraint, the data of eight participants could not have been considered in the analyses.

### Results and Discussion

#### Hit Rate

The independent-measures t-test on the percentage of correctly recalled POIs did not show a significant difference in the means for 2D-GRIDS (M = 82.3, SD = 13.6) and 3D-GRIDS (M = 84.1, SD = 10.5; t(58) = -0.57, p = .57).

#### Spatial Accuracy

**Fig 3:**
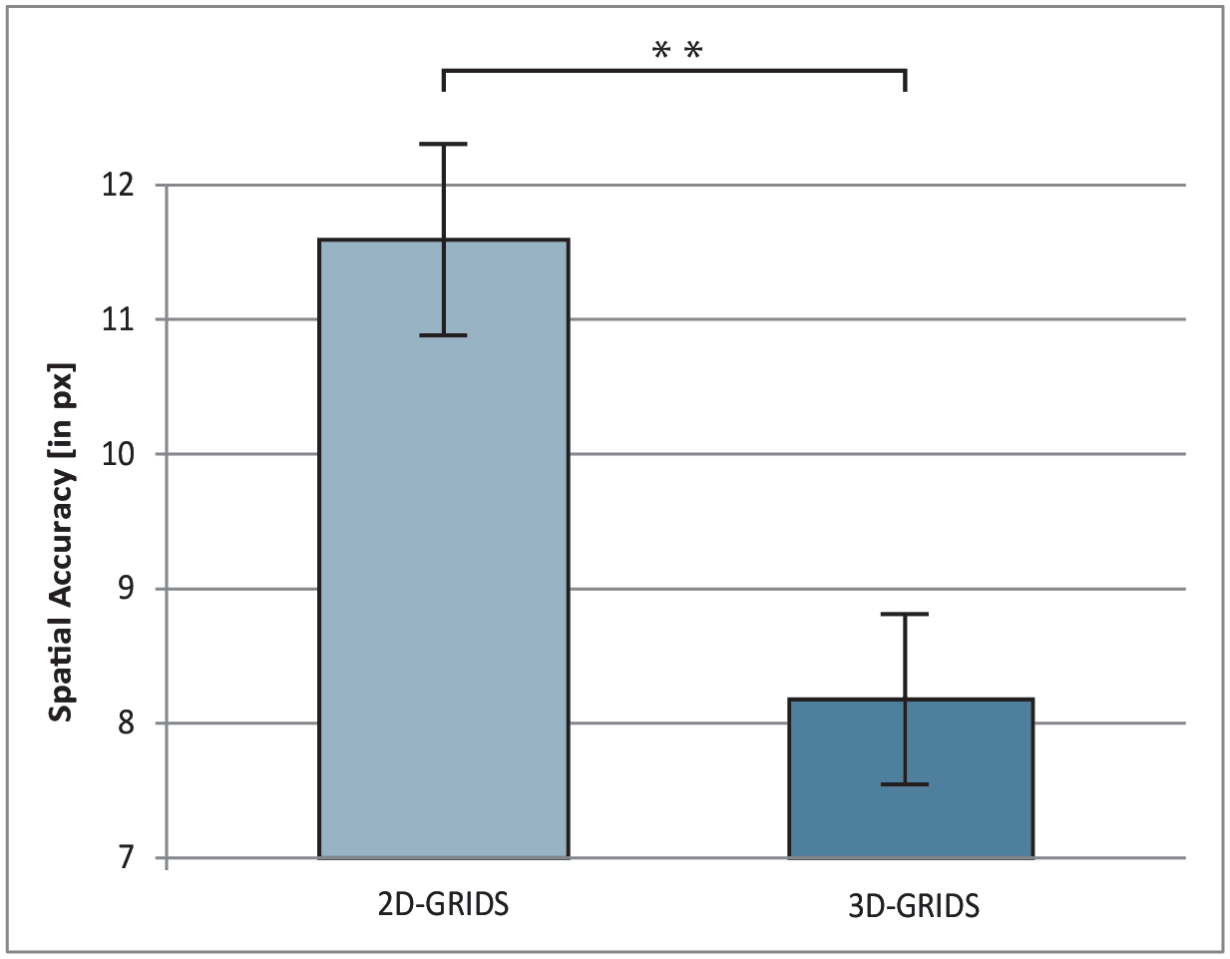
Spatial Accuracy differences between 2D-GRIDS and 3D-GRIDS. Spatial Accuracy refers to the mean distances between the original and recalled places of interest (POIs). The recall of a POI was considered as correct if the recall location fell within a linear distance of 0–40.27 px (0–1 cm). Error bars represent the standard error of the mean. ** = p <. 01.

The independent-measures t-test on the mean distances of correctly recalled POIs revealed a significant difference in the means for 2D-GRIDS (M = 11.6, SD = 3.8) and 3D-GRIDS (M = 8.2, SD = 3.5); t(58) = 3.61, p = .001) (Fig. 3).

The results of this study show the potential of elevating grid structures in maps for human object location memory. Projecting an overlaying map grid closer to the user than other information layers increases the recall accuracy of object locations. In accordance with previous research [52, 53, 56, 57], content highlighted in True-3D attracts and increases the user’s visual attention. An increased attention to the regular and orthogonal grid structures seems to further improve the chunking effect of grids that might lead to a more accurate object location memory (see also [32–35, 39, 40]).

The present study further indicates that True-3D elevated grids do not significantly affect the number of object locations that can be encoded and recalled correctly. Only a non-significant tendency indicates an advantage of the 3D over the 2D condition concerning the hit rates.

In addition to grids, previous studies also reported effects of linear map elements on perception and memory, such as boundary lines, rivers or streets [16, 30, 31, 40, 66]. In contrast to grids, these elements represent content which is a fundamental part of the map theme. Therefore, they are expected to have a higher intrinsic attraction for the map reader than the grid pattern which is rather neutral for the map theme. We are not aware of any other study varying the accentuation of such intrinsic map elements in True-3D. Therefore, we carried out an additional examination of the effects of True-3D accentuated streets in urban topographic maps. If a similar improvement of object location memory was visible, based on a True-3D depiction of linear but non-regular map content, this would clearly support our assumption that True-3D accentuated map elements attract attention that guides object location memory performance.

## Study 2: 3D vs. 2D Streets

### Methods

**Fig 4:**
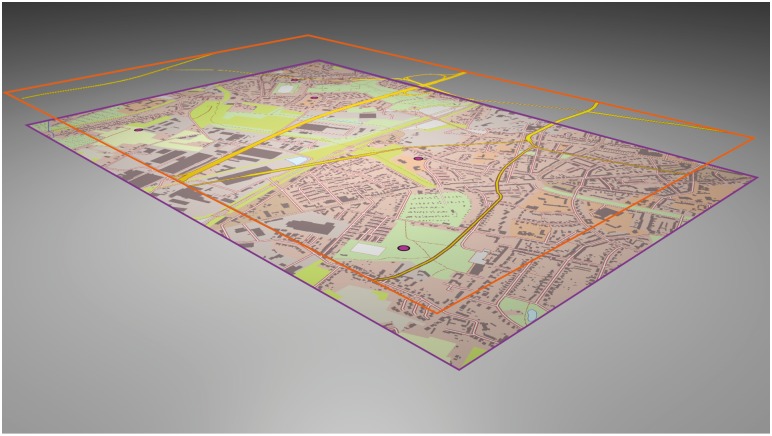
Oblique bird’s eye view on a study-test map, incl. True-3D accentuated streets. This figure illustrates the layer arrangement in two different elevation levels. The first level represents the topographic base map (violet frame; cf. map of Herne, Fig. 1), The second level indicates the overlaying streets (orange frame). Both layers were projected in a 30 cm*20 cm format. The depth offset between both layers was 5 cm. The five point symbols in Layer 1 represent the randomly selected places of interest (POIs) to be learned and recalled.

The methods reported in the first study were replicated in the second study. The focus of this experiment was not on the effects of grids, but on True-3D accentuated highways and main streets in highly urban maps. Fig. 4 shows one of the study-test maps, including the True-3D accentuated streets. In contrast to grids, streets are content-related linear map features that provide a rather arbitrary orientation pattern. Previous studies showed that linear map features influence spatial memory tasks and can be of advantage for human object location memory [8,18,19,63]. In this study, each map included 9 or 10 street-based sub-regions on the map surface. On an average, each sub-region comprised an area of 64.5 cm². A new set of 50 randomly distributed POIs was selected for the maps used in this study.

Seventy-six new participants (36 female, 40 male) aged between 19 and 33 [M = 23.9; SD = 2.9] took part in the study for pay. As it was defined that at least one object had to be recalled correctly per map, the data of ten participants were not considered in the statistical analyses. The between-subjects design included the factor STREETS (3D/2D). Due to the availability of data, this study included a total of eight highly urban maps as study materials, four in 3D and four in 2D. One map of each condition was used in the practice trial, the other three maps for the study-test trials. These three maps represented the topographies of Gelsenkirchen, Hagen and Herne (see Fig. 1). The average number of distinct objects in the maps was 5,097 (DOs). The average object density was 8.50 (DOs/cm²).

### Results and Discussion

#### Hit Rate

**Fig 5:**
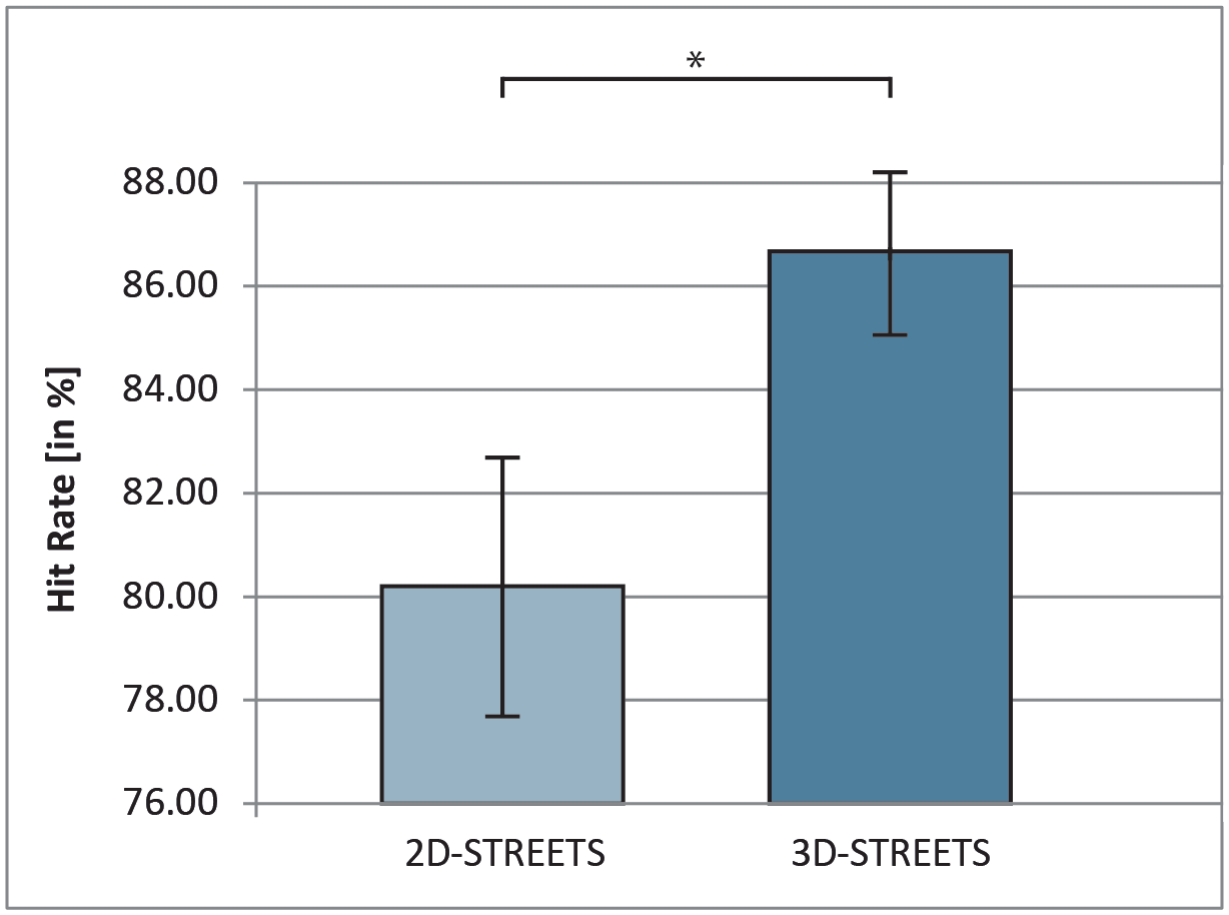
Hit Rate differences between 2D-STREETS and 3D-STREETS. Hit Rate refers to the mean percentage of correctly recalled places of interest (POIs). The recall of a POI was considered as correct if the recall location fell within a linear distance of 0–40.27 px (0–1 cm). Error bars represent the standard error of the mean. * = p <. 05.

The independent-measures t-test on the percentage of correctly recalled POIs showed a significant difference in the means for 2D-STREETS (M = 80.2, SD = 14.7) and 3D-STREETS (M = 86.7, SD = 9.9); t(64) = -2.10, p = .04 (Fig. 5).

#### Spatial Accuracy

The independent-measures t-test on the mean distances of correctly recalled POIs did not reveal a significant difference in the means for 2D-STREETS (M = 11.3, SD = 4.3) and 3D-STREETS (M = 9.6, SD = 4.1); t(64) = 1.60, p = .12).

The results of this study show that, compared to a 2D representation, the True-3D depiction of streets in urban topographic maps significantly increases the number of object locations that map users can recall correctly. Moreover, a trend is indicated that the True-3D variety also leads to more accurate results. It seems likely that the attraction of visual attention caused by the True-3D accentuated map elements increases the likelihood of these elements to guide object location memory (see also [49, 52, 53, 56, 57]). The True-3D depiction of streets emphasises their structure on the map surface, which seems to provide users with a better spatial orientation pattern to encode and recall object locations, compared to a 2D counterpart. This optimised spatial orientation pattern is associated with an emphasis of content-related linear chunks, which were previously suggested as graphic structures improving spatial memory performance [16, 30, 31, 40, 66].

## General Discussion

The present set of experiments was designed to investigate the effects of True-3D accentuated linear map elements in a human object location memory paradigm. Both, grids (study 1) and streets (study 2) that were projected (5 cm) closer to the viewer influenced the object location memory performance, in support of our initial hypotheses. This clearly extends previous findings of improved information processing tasks due to 3D depth effects [39]. Previous studies on stereoscopic 3D content showed that information projected closer to the viewer attracts attention and is processed faster [52, 53, 55, 56]. Both studies reported in this paper confirmed the positive impact of True-3D accentuation for information processing, and show the potential of True-3D as modern cartographic design option to increase object location memory.

Study 1 showed strong advantages for the 3D- over the 2D-GRIDS in terms of spatial accuracy (see Fig. 3), but no effects were found concerning hit rates. The opposite result pattern was observed in study 2. 3D-STREETS improved the hit rate but not the spatial accuracy (see Fig. 5).

The strong increase of spatial accuracy observed in study 1 makes us believe that the additional accentuating of a regular grid pattern in True-3D clearly influences the way map information is perceived, learned and recalled. Previous studies already provided evidence that grids overlaid on 2D maps or map-like visualisations helped to improve the performance of object location memory [30, 33, 40]. The aligned shape of grids is a graphic pattern people have a lot of practice and experience with [67]. The orientation along a grid pattern in a map is comparable to reading a graph in a coordinate system, a task that requires the reader’s accuracy [68]. It seems apparent that the orthogonal shape of a grid addresses a visual routine of the reader that is directed to a more accurate task solving. Highlighting this regular shape by elevating it seems to attract additional attention to this orthogonal scheme. In previous studies, it was also demonstrated that modifications of single grid design parameters, such as grid line spacing or grid opacity, has an impact on the accuracy of object location recall [39, 69].

Moreover, in a previous study it was shown that, compared to a ‘no grid’-condition, an additional layer of grid lines generally supports both, hit rate and spatial accuracy [30]. The supportive impact of grids on the hit rate measure however decreases in maps of high visual details [30]. If grid design is changed in different complex maps, such as spacing grid lines close to each other, the hit rate performance decreases [39]. It seems likely that, due to the general high amount of visual details in urban topographic maps, the True-3D depiction of the orthogonal grid pattern does not bring any further advantages for the hit rate measure.

In contrast to the first study, study 2 did not include any artificial and regular linear elements regionalising the map surface, such as grids. In general, the effect on the hit rate measure (see Fig. 5) indicates that a regionalisation of the map surface based on arbitrarily running linear elements, such as streets or boundaries, improves spatial memory (see also [8,12,16,18,19,29,63]). The number of map objects that are recalled correctly can apparently be increased by highlighting the street courses in True-3D. At the same time, this alteration of streets in depth seems not be sufficient enough to be a visualisation tool for the improvement of the spatial accuracy in object location memory.

The reference pattern in study 2 was made up of the content-related layer of streets that divides up the map into several sub-regions of irregular course, shape and size. Although grids and streets are both represented lines, they apparently underlie different conceptual factors in map-reading [68]. Based on the present results, it seems likely that it is the regular pattern of grids which has the greater impact on spatial accuracy, whereas irregular streets emphasize overall hit rates. It needs to be discussed whether the regularity of GRIDS specifically supports the encoding of coordinate information, whereas irregular patterns like streets in particular lead to a breaking-up or regionalization of the spatial layout. Such content-related linear spatial information in True-3D with its higher semantic importance for the map might signal attraction to the map reader and seems to be particularly helpful for encoding correct object locations, but without effects on spatial accuracy. Thus, one could speculate that an irregular pattern in True-3D is not as sufficient as a regular GRID in True-3D to provide detailed coordinate cues with high spatial accuracy.

Of particular note is that the average number and length of streets forming the sub-regions in study 2 were smaller than the average number and length of grid lines in study 1. As a consequence, each street-based sub-region in study 2 covered a larger area of the map surface (grid regions: Ø 25 cm²—street regions: Ø 64.5 cm²). Hence, a main difference between both studies is likely due to the fact that street lines in study 2, on average, have been in greater distance from the randomly distributed POI locations than the grid lines in study 1. Thus, shape and regularity of the reference frames accentuated by True-3D visualisation differ between both studies, and thus could potentially explain the differences in the result patterns of these studies. In a previous study [39], in which we compared a grid line spacing of less density with the present 5 cm spacing interval, no differences in hit rates but a lower spatial accuracy was determined for the less dense condition in topographic maps. A lower spatial accuracy based on the relative distance from the reference frame in study 2 might well explain why no effect was observed in the spatial accuracy measure.

This is the first study documenting advantages of True-3D visualisation for map-based object location memory. Of particular interest is that the True-3D accentuating of grids and streets extends the effects of 2D grids in highly urban topographic maps. Previous studies documented that the size of the grid effect in highly urban topographies, i.e. in maps with high amounts of details and objects, decreases [30]. Thus, if a cartographer decides to overlay a map with grids, the spatial accuracy of the user’s object location can be further increased by True-3D emphasis. If grids are not used (e.g. for aesthetic reasons [70]), the True-3D accentuating of the dominant streets still leads to an improvement of the amount of information that is encoded and recalled in object location memory.

## Conclusion

The modern cartographic design option to locate specific map elements at different positions in depth brings advantages for human object location memory. This project shows that the True-3D depiction of linear map features in highly urban topographic maps (scale 1/10,000), such as street courses and grids, can improve the hit rate or the spatial accuracy of recalled object locations. The spatial accuracy of a mental image of a grid-featured urban topographic map can be increased by visualising the overlaying grid construction closer to the map user. Alternatively, if no grid construction is overlaid on an urban topographic map, the True-3D accentuation of the dominant street representations allows the map user to memorise and recall more object locations. These results show the potential of True-3D as a design option that can help to improve cartographic communication and the quality of cognitive representations of map information. Future studies will focus on the identification of specific design parameters for True-3D cartographic media that enhance spatial memory.

## Supporting Information

S1 Data Raw data of study 1. (SAV)

S2 Data Raw data of study 2. (SAV)

S3 Data Raw data of study 1. (XLS)

S4 Data Raw data of study 2. (XLS)

## References

[1] Tversky B (1993) Cognitive maps, cognitive collages, and spatial mental models In: Frank AU, Campari I, editors. Spatial information theory: a theoretical basis for GIS. Berlin: Springer pp. 14–24.

[2] Meneghetti C, Ronconi L, Pazzaglia F, De Beni R (2013) Spatial mental representations derived from spatial descriptions: the predicting and mediating roles of spatial preferences, strategies, and abilities. Brit J Psychol 105: 295–315. 10.1111/bjop.12038 25040003

[3] Brunyé TT, Taylor HA (2008) Extended experience benefits spatial mental model development with route but not survey descriptions. Acta Psychologica 127: 340–354. 17723221 10.1016/j.actpsy.2007.07.002

[4] Daniel M, Denis M (1998) Spatial descriptions as navigational aids: a cognitive analysis of route directions. Kognitionswissenschaft 7: 45–52.

[5] Dickmann F (2012) City maps versus map-based navigation systems—an empirical approach to building mental representations. Cartogr J 49: 62–69.

[6] Wolbers T, Hegarty M (2010) What determines our navigational abilities? Trends Cogn Sci 14: 138–146. 10.1016/j.tics.2010.01.001 20138795

[7] Bitter R (1999) Kognitive Karten und Kartographie. Kartographische Nachrichten 49: 93–97.

[8] Dickmann F, Edler D, Bestgen A, Kuchinke L (2013) Spatial distortions in cognitive maps—a chance and challenge to enrich the principles of map design. Kartographische Nachrichten 63: 174–181.

[9] Montello DR (1998) Kartenverstehen: Die Sicht der Kognitionspsychologie. Zeitschrift für Semiotik 20: 91–103. 10.1107/S0108767309007235 19349661

[10] MacEachren AM (1991) The role of maps in spatial knowledge acquisition. Cartogr J 28: 152–162.

[11] Lloyd R (1989) Cognitive maps: encoding and decoding information. Ann Assoc Am Geogr 77: 191–207.

[12] Hommel B, Gehrke J, Knuf L (2000) Hierarchical coding in the perception and memory of spatial layouts. Psychol Res 64: 1–10. 11109863 10.1007/s004260000032

[13] McNamara TP, Hardy JK, Hirtle SC (1989) Subjective hierarchies in spatial memory. J Exp Psychol Learn Mem Cogn 15: 211–227. 2522511 10.1037//0278-7393.15.2.211

[14] Hirtle S, Jonides J (1985) Evidence of hierarchies in cognitive maps. Mem Cognit 13: 208–217. 4046821 10.3758/bf03197683

[15] Stevens A, Coupe P (1978) Distortions in judged spatial relations. Cogn Psychol 10: 422–437. 699514 10.1016/0010-0285(78)90006-3

[16] Eastman JR (1985) Cognitive models and cartographic design research. Cartogr J 22: 95–101.

[17] Coren S, Girgus JS (1980) Principles of perceptual organization and spatial distortion: the gestalt illusions. J Exp Psychol Hum Percept Perform 6: 404–412. 6447756 10.1037//0096-1523.6.3.404

[18] Klippel A, Knuf L, Hommel B, Freksa C (2004) Perceptually induced distortions in cognitive maps. Spatial Cognition IV: Reasoning, Action, Interaction: 204–213.

[19] Hurts K (2005) Common Region and Spatial Performance Using Map-like Displays. Proceedings of the Human Factors and Ergonomics Society Annual Meeting: 1593–1597.

[20] Tversky B, Schiano DJ (1989) Perceptual and conceptual factors in distortions in memory for graphs and maps. J Exp Psychol Gen 118: 387–398. 2531198 10.1037//0096-3445.118.4.387

[21] McNamara TP (1986) Mental representations of spatial relations. Cogn Psychol 18: 87–121. 3948491 10.1016/0010-0285(86)90016-2

[22] Okabayashi H, Glynn SM (1984) Spatial cognition: systematic distortions in cognitive maps. J Gen Psychol 111: 271–279. 6512518 10.1080/00221309.1984.9921116

[23] Fabrikant SI, Lobben A (2009) Cognitive issues in geographic information visualisation. Cartographica 44: 139–143.

[24] Tversky B (1981) Distortions in memory for maps. Cogn Psychol 13: 407–433.

[25] Oliva A, Torralba A (2006). Building the gist of a scene: the role of global image features in recognition. Prog Brain Res 155: 23–36. 17027377 10.1016/S0079-6123(06)55002-2

[26] Henderson JM (2003) Human gaze control during real-world scene perception. Trends Cogn Sci 7: 498–504. 14585447 10.1016/j.tics.2003.09.006

[27] Biederman I, Mezzanotte RJ, Rabinowitz JC (1982) Scene perception: detecting and judging objects undergoing relational violations. Cogn Psychol 14: 143–177. 7083801 10.1016/0010-0285(82)90007-x

[28] Eastman RJ (1985) Graphic organization and memory structures for map learning. Cartographica 22: 1–20.

[29] Clements-Stephens AM, McKell-Jeffers GO, Maddux J-M, Shelton AL (2011) Strategies for spatial organization in adults and Children. Vis cogn 19: 886–909.

[30] Edler D, Bestgen A-K, Kuchinke L, Dickmann F (2014) Grids in topographic maps reduce distortions in the recall of learned object locations. PLoS One 9(5): e98148 10.1371/journal.pone.0098148 24869486 PMC4037198

[31] Kulik L, Klippel A (1999) Reasoning about cardinal directions using grids as qualitative geographic coordinates In Freksa C, Mark DM, editors. Spatial Information Theory, Berlin: Springer pp. 205–220.

[32] Stainer MJ, Scott-Brown KC, Tatler BW (2013) Behavioral biases when viewing multiplexed scenes: scene structure and frames of reference for inspection. Front Psychol 4: 624 10.3389/fpsyg.2013.00624 24069008 PMC3781347

[33] Leifert S (2011) The influence of grids on spatial and content memory. Proceedings of the 2011 annual conference extended abstracts on Human factors in computing systems: 941–946.

[34] Martin R, Houssemand C, Schiltz C, Burnod Y, Alexandre F (2008) Is there continuity between categorical and coordinate spatial relation coding? Evidence from a grid/no-grid working memory paradigm. Neuropsychologia 46: 576–594. 18037455 10.1016/j.neuropsychologia.2007.10.010

[35] Wolfe JM, Võ ML-H, Evans KK, Greene MR (2011) Visual search in scenes involves selective and nonselective pathways. Trends Cogn Sci 15: 77–84. 10.1016/j.tics.2010.12.001 21227734 PMC3035167

[36] Hake G, Grünreich D, Meng L (2002) Kartographie. 8th ed. Berlin: de Gruyter 604 p.

[37] Robinson AH, Morrison JL, Muehrcke PC, Kimerling AJ, Guptill SC (1995) Elements of cartography. 6th ed. New York: Wiley 688 p.

[38] Maling DH (1992) Coordinate systems and map projections. 2nd ed. Oxford: Pergamon 476 p.

[39] Edler D, Bestgen A-K, Kuchinke L, Dickmann F (2014) The effects of grid line separation in topographic maps for object location memory. Cartographica (accepted).

[40] Bestgen A, Edler D, Dickmann F, Kuchinke L (2013) Grid or no grid: distance distortion in recognizing spatial information from complex cartographic maps. Proceedings of CogSci 2013–35^th^ Annual Meeting of the Cognitive Science Society, (Berlin, Germany). MindModeling@Home website. Available: http://mindmodeling.org/cogsci2013/papers/0062/paper0062.pdf. Accessed 2014 May 5. 10.1364/AO.53.007898 25607866

[41] Dickmann F (2010) The potential of the lenticular foil technique for thematic cartography. Cartogr J 47: 250–256. 10.1016/j.ijbiomac.2010.04.014 20434482

[42] Buchroithner MF (2007) Echtdreidimensionalität in der Kartographie: Gestern, heute und morgen. Kartographische Nachrichten 57: 239–248.

[43] Knust C, Buchroithner MF (2014) Principles and terminology of true-3D geovisualisation. Cartogr J 51: 191–202.

[44] Buchroithner MF (2012) True-3D in cartography Autostereoscopic and solid visualisation of geodata. Berlin: Springer 300 p.

[45] Kraak M-J (1993) Three-dimensional map design. Cartogr J 30: 188–194.

[46] Holliman NS, Dodgson NA, Favalora GE, Pockett L (2011) Three-dimensional displays: a review and application analysis. IEEE Transactions and Broadcastings 57: 362–371.

[47] Bröhmer K, Knust C, Dickmann F, Buchroithner MF (2013) Z-Axis based visualization of map elements—cartographic experiences with 3D monitors using lenticular foil technology. Cartogr J 50: 211–217.

[48] Dickmann F, Dodt J, Schmidt B (2009) Zum Potenzial der Lentikulartechnik in der thematischen Kartographie. Kartographische Nachrichten 59: 295–302.

[49] Wood D (1968) Visual perception and map design. Cartogr J 5: 54–64.

[50] Lang C, Nguyen TV, Katti H, Yadati K, Kankanhalli M et al (2012) Depth matters: influence of depth cues on visual saliency In Fitzgibbon A, Lazebnik S, Perona P, Sato Y, Schmid C, editors: Computer Vision—ECCV 2012, Florence pp. 101–115. 10.1113/jphysiol.2014.284919

[51] Huynh-Thu Q, Barkowsky M, Le Callet P (2011) The importance of visual attention in improving 3D-TV viewing experience: overviews and new perspectives. IEEE Transactions on Broadcasting 57: 421–431.

[52] Häkkinen J, Kawai T, Takatalo J, Mitsuya R, Nyman G. (2010) What do people look at when they watch stereoscopic movies? In Woods AJ, Holliman NS, Dodgson NA, editors: Proceedings of SPIE 7524, Stereoscopic Displays and Applications XXI: 75240E.

[53] Jansen L, Onat S, König P. (2009) Influence on disparity on fixation and saccades in free viewing of natural scenes. Journal of Vision, 9 (1:29): 1–19. 10.1167/9.1.29 19271899

[54] Kollmorgen S, Nortmann N, Schröder S, König P (2010) Influence of low-level stimulus features, task dependent factors, and spatial biases on overt visual attention. PLoS Comput Biol 6(5): e1000791 10.1371/journal.pcbi.1000791 20502672 PMC2873902

[55] Huynh-Thu Q, Schiatti L (2011) Examination of 3D visual attention in stereoscopic video content In Rogowitz BE, Pappas TN, editors: Proceedings of SPIE 7865, Human Vision and Electronic Imaging XVI: 78650J.

[56] Wolfe J, Horowitz T (2004) What attributes guide the deployment of visual attention and how do they do it? Natural Reviews Neuroscience 5: 495–501. 15152199 10.1038/nrn1411

[57] Edler D, Huber O, Knust C, Buchroithner M, Dickmann F (2014b) Spreading map information over different depth layers—an improvement for map-reading efficiency? Cartographica 49: 153–163.

[58] Tavanti M, Lind M. (2001) 2D vs 3D, implications on spatial memory. In: Proceedings of the IEEE Symposium on Information Visualization: 7 p.

[59] Cockburn A, McKenzie B (2004) Evaluating spatial memory in two and three dimensions. Int J Hum-Comput St 61: 359–373.

[60] Lloyd RE, Bunch RL (2008) Explaining map-reading performance efficiency: gender, memory, and geographic information. Cartogr Geogr Inf Sci 35: 171–202.

[61] Montello DR, Sullivan CN, Pick HL (1994) Recall memory for topographic and natural terrain: effects of experience and task performance. Cartographica 31: 18–36.

[62] Tversky B (1992) Distortions in cognitive maps. Geoforum 23: 131–138.

[63] McNamara TP, Ratcliff R, McKoon G (1984) The mental representation of knowledge acquired from maps. J Exp Psychol Learn Mem Cogn 10: 723–732. 6239008 10.1037//0278-7393.10.4.723

[64] Baatz M, Schaepe A (2000) Multiresolution segmentation—an optimization approach for high quality multi-scale image segmentation In: Strobl J, Blaschke T, Griesebner G, editors. Angewandte Geographische Informationsverarbeitung XII. Karlsruhe: Wichmann pp. 12–23.

[65] Richards W (1971) Anomalous stereoscopic depth perception. J Opt Soc Am 61: 410–414. 5542548 10.1364/josa.61.000410

[66] Ooms K (2012): Maps, how do users see them? An in depth investigation of the map users’ cognitive processes PhD-Thesis, Ghent University, Ghent.

[67] Kosslyn SM (1989): Understanding charts and graphs In: Appl Cognitive Psych, 3, pp. 185–226.

[68] Tversky B, Schiano DJ (1989) Perceptual and conceptual factors in distortions in memory for graphs and maps. J Exp Psychol Gen 118: 387–398. 2531198 10.1037//0096-3445.118.4.387

[69] Edler D, Bestgen A-K, Kuchinke L, Dickmann F (2014) Gitter als graphisches Korrektiv im Aufbau mentaler Raumrepräsentationen—Eine empirische Fallstudie zur Opazität von Gitterkreuzen in Topographischen Karten. Kartographische Nachrichten 64: 243–251.

[70] Kent AJ (2013) From a dry statement of facts to a thing of beauty: understanding aesthetics in the mapping and counter-mapping of place. Cartographic Perspectives 73: 39–60.

